# 

**Published:** 1889-10

**Authors:** 


### *SPECIAL.*

We desire to call attention to the Premiums which we offer to new subscribers to our Journal. They have been selected with less regard to cost than to their usefulness.

Bear in mind that only those subscribers who remit directly to this office are entitled to them. This is not always understood. The great increase in the circulation of the Journal during the past two years enables us to keep pace with the foremost publications in the land in dealing with our patrons, a feature which, we are glad to say, is not unappreciated.

### BOOK NOTICES.

THE CENTURY has in preparation a series of papers on topics relating to The Gold Hunters of California. The articles will be prepared for the most part, as were the War Papers, by prominent participants in the events which they describe; and they will include accounts of Early Explorations, Life in California before the Gold Discovery, the Finding of Gold in 1848 at Sutter,s Fort, the Journey to California by the different routes (around the Horn, across the plains, by Nicaragua› and by Panama), Life in the Mining Camps and in San Francisco, and other important aspects of California life at the time. These papers wiil present many interesting aspects of the pioneer period, its romance and adventure, its tragedy and pathos, and its poetry and humor. The publication of the papeıs will be delayed until the series if further advanced.

LITERARY.—The *National Magazine* is the name of a new literary venture of Chicago, which begins with the October number. It is published under the auspices of the new "National Uuiversity," which opens October 1st, of which it is the organ. The first number will contain articles on literary, educational and scientific subjects, and a prospectus of the University, which is said to be modelled after the London University and has extensive non-resident courses, teaching many subjects by mail. Published at 128 Clark Street.

INEBRIETY, its Etiology, Pathology, Treatment and Jnrisprudence, by Norman Kerr, M. D., F. L. S. London, Eng. Second edition.—A very brief mention of this exhaustive treatise was given in our September issue, but its high merit entitles it to a much more extended notice. The book embraces nearly 500 pages and treats of all forms of inebriety and their effect upon both body and mind, not only of the inebriate but also of his progeny, with the most approved methods of treatment and cure. There are marginal catch-notes, making easy references to subjects. Take it all in all, this work is oue of very great value, especially to physicians and would-be experts in the various phases of inebriety, alcoholism, demntia and insanity.

THE OCTOBER CENTURY, among other matters of unusual interest, contains an illustraled treatise on Base Ball, which brings it within a popular craze and insures for it the favor of our athletic wielders of the bat.

WE ARE under obligations to the several authors for the following pamphlets and treatises: To Professor Fessenden N. Otis, M. D., "Résumé of Seventeen Years' Practice in Special Cases," etc.; "Seventeenth Annual Meeting, American Health Association of Brooklyn, N. Y. To Walbridge & Co., 17–23 Vanderwater Street, for a fancifully illustrated almanac for 1890. It is very complete and elegantly brought out.

PAMHLETS RECEIVED. The Senses, Five or Seven, by Wm. M. Mc Laury M. D. Report of Yale Obesrvatory for 1888-9. August Bulletin, Agricultural Department-Cornell University.

ENSWORTH MEDICAL COLLEGE, ST. JOSEPH, MO., Thirteenth Annual Announcement, Session of 1889–90.

